# Factors associated with willingness to wear an electronic overdose detection device

**DOI:** 10.1186/s13722-019-0153-5

**Published:** 2019-07-03

**Authors:** Keith Ahamad, Huiru Dong, Cheyenne Johnson, Kanna Hyashi, Kora DeBeck, M. J. Milloy, Evan Wood

**Affiliations:** 1 British Columbia Centre on Substance Use, Vancouver, Canada; 2 Department of Family Medicine, University of British Columbia, Vancouver, Canada; 3 Department of Medicine, University of British Columbia, Vancouver, Canada; 4 School of Public Policy, Simon Fraser University, Burnaby, Canada; 5 Faculty of Health Sciences, Simon Fraser University, Burnaby, Canada; 6 St. Paul’s Hospital, 553-1081 Burrard Street, Vancouver, BC V6Z 1Y6 Canada

**Keywords:** Addiction, Willingness to wear, Overdose detection device

## Abstract

**Background:**

North America is in the midst of an opioid overdose epidemic. Although take-home naloxone and other measures have been an effective strategy to reduce overdoses, many events are unwitnessed and mortality remains high amongst those using drugs alone. While wearable devices that can detect and alert others of an overdose are being developed, willingness of people who use drugs to wear such a device has not been described.

**Methods:**

Drug using persons enrolled in a community-recruited cohort in Vancouver, Canada, were asked whether or not they would be willing to wear a device against their skin that would alert others in the event of an overdose. Logistic regression was used to identify factors independently associated with willingness to wear such a device.

**Results:**

Among the 1061 participants surveyed between December 2017 and May 2018, 576 (54.3%) were willing to wear an overdose detection device. Factors independently associated with willingness included ever having overdosed (adjusted odds ratio [AOR] = 1.39, 95% confidence interval [CI] 1.06–1.83), current methadone treatment (AOR = 1.86, 95% CI 1.45–2.40), female gender AOR = 1.41, 95% CI 1.09–1.84) and a history of chronic pain (AOR = 1.53, 95% CI 1.19–1.96). Whereas homelessness (AOR = 0.67, 95% CI 0.50–0.91) was negatively associated with willingness.

**Conclusions:**

A high level of willingness to wear an overdose detection device was observed in this setting and a range of factors associated with overdose were positively associated with willingness. Since some factors, such as homelessness may be a barrier, further research is needed to investigate explanations for unwillingness and to evaluate real world acceptability of a wearable overdose detection devices as this technology becomes available.

## Background

Across North America opioid overdose deaths have emerged as a major public health concern. In 1999, the U.S. age adjusted death rate from opioid overdoses was 6.1 per 100,000 standard population and by 2015 increased to 16.3 [1]. In more recent years, a number of North American settings, and increasingly elsewhere, have seen a spike in overdoses and overdose deaths due to the introduction of illicitly manufactured fentanyl in the illicit drug supply. For example, in British Columbia, Canada the illicit drug overdose death rate in 2017 was 30.2 per 100,000 population with fentanyl or its analogues detected in more than 80% of deaths [2, 3].

In efforts to prevent illicit drug related overdose deaths, harm reduction initiatives have been expanded including “take home naloxone” (THN) programs and other overdose prevention interventions [4]. Early estimates of THN programs have suggested they have proven successful at reducing overdose deaths [5, 6]; however, death rates in these settings remains unacceptably high [7].

To this end, wearable overdose detection technology has emerged as an active area of research [4] as it has been suggested that it may play a role in this population by “detecting an impending overdose” and send a signal for help or even administer naloxone for overdose reversal [8]. A recent study demonstrated that cell phone technology using short-range active sonar technology was able to identify respiratory depression, apnea, and gross motor movements associated with acute opioid toxicity [9]. However, since little is known about the willingness of drug users to wear such a device, we undertook this study amongst those participating in a cohort study in Vancouver, Canada to examine willingness to wear a device while using drugs.

## Methods

Data for this study were derived from the Vancouver Injection Drug Users Study (VIDUS), an open prospective cohort of HIV-seronegative individuals who inject drugs, AIDS Care Cohort to Evaluate Access to Survival Services (ACCESS), an open prospective cohort of HIV-seropositive individuals who use illicit drugs, and the At-Risk Youth Study (ARYS), a multi-year study of street-involved youth in Vancouver, Canada. Detailed methodology has previously been described [10, 11]. Briefly, participants were eligible for the study if they were 18 years or older of age, used illicit drugs other than cannabis within the past month, resided in the Greater Vancouver Region, and provided informed consent. Participants were recruited through extensive street-based outreach methods and snowball sampling, beginning in May 1996. At baseline and every 6 months thereafter, participants completed an interviewer-administered questionnaire that elicited information regarding socio-demographic characteristics, drug use, HIV risk behaviours and treatment utilization and underwent an examination by a nurse. Participants received $30 CAD stipend for each visit. VIDUS and ACCESS studies recruitment and follow up procedures are essentially identical with the exception of questions specific to HIV infection so as to enable merged analyses. Both the VIDUS and ACCESS studies were ethically approved by the Research Ethics Board of Providence Health Care/University of British Columbia.

For the present analyses, we assessed whether participants were willing to wear a device to detect overdose by adding questions to follow up visits between December 2017 and May 2018. Specifically, within the main questionnaire participants were asked: “Researchers are developing a medical device that would alert others if you were having an opiate overdose. Would you be willing to wear a small device against the skin on your chest while you are using drugs?” Participants who answered, “Yes” were compared to those who answered “No”/“Unsure” on a variety of a priori selected socio-demographic, behavioral and drug use variables hypothesized to be associated with willingness to wear a device. Since such a device was not available in Canada at the time these questions were utilized, staff were trained to answer general questions about the device describing it as above as the size of a phone.

These variables included: ethnicity (Caucasian vs. other); female gender (yes vs. no); age (per year older); daily heroin injection (yes vs. no); daily cocaine injection (yes vs. no); daily crack smoking (yes vs. no); ever had a non-fatal overdose (yes vs. no); homelessness (yes vs. no); methadone treatment (yes vs. no); chronic pain (yes vs. no); HIV seropositivity (yes vs. no). Unless otherwise noted, all drug use related variables refer to the 6-month period prior to the interview. All variable definitions have been used extensively and were identical to earlier publications [12, 13].

As a first step, bivariable logistic regression analyses were used to determine factors associated with the willingness to wear a device. To identify factors that were independently associated with our outcome of interest, variables significant at the *p *< 0.10 threshold in bivariable analyses were entered in a multivariable logistic regression model. Using the backwards selection procedure, we constructed the final multivariate model with the best fit, as indicated by the lowest AIC value. All statistical analyses were performed using the SAS software version 9.4 (SAS, Cary, NC, USA). All *p* values are two sided.

## Results

Between December 2017 and May 2018, 1061 opioid using VIDUS, ACCESS and ARYS participants answered the relevant question regarding potential device use and were interviewed and included in the present analysis. Among these individuals, median age was 44.2 (Inter-quartile range [IQR]: 31.3–53.9), 391 (36.9%) were female and 615 (58.0%) were Caucasian. In comparison to those study participants who were included in the present study, those excluded (n = 365) were more likely to be older in age (median 49.3 [IQR: 33.4–57.0]; *p* value < 0.001), but there was no significant difference regarding gender (*p* value = 0.963) and ethnicity (*p* value = 0.262). The characteristics of the study sample stratified by willingness to wear a device are presented in Table 1.

**Table 1:** Characteristics of study participants assessed for willingness to wear an overdose detection device (n = 1061)

Variables	Value	Total n (%)	Willingness = yes (n = 576)	Willingness = no/unsure (n = 485)
Ethnicity	Caucasian	615 (58.0)	329 (57.1)	286 (59.0)
	Other	445 (41.9)	246 (42.7)	199 (41.0)
Gender	Female	391 (36.9)	237 (41.1)	154 (31.8)
	Male	670 (63.1)	339 (58.9)	331 (68.2)
Age (years)	Median (Q1–Q3)	44.2 (31.3–53.9)	45.4 (33.0–54.0)	42.5 (29.6–53.9)
Daily heroin use^a^	Yes	336 (31.7)	196 (34.0)	140 (28.9)
	No	725 (68.3)	380 (66.0)	345 (71.1)
Daily cocaine use^a^	Yes	34 (3.2)	18 (3.1)	16 (3.3)
	No	1027 (96.8)	558 (96.9)	469 (96.7)
Daily crack use^a^	Yes	99 (9.3)	55 (9.5)	44 (9.1)
	No	962 (90.7)	521 (90.5)	441 (90.9)
Ever overdosed	Yes	748 (70.5)	425 (73.8)	323 (66.6)
	No	311 (29.3)	150 (26.0)	161 (33.2)
Homelessness^a^	Yes	239 (22.5)	109 (18.9)	130 (26.8)
	No	822 (77.5)	467 (81.1)	355 (73.2)
Methadone treatment^a^	Yes	472 (44.5)	299 (51.9)	173 (35.7)
	No	588 (55.4)	277 (48.1)	311 (64.1)
Chronic pain	Yes	556 (52.4)	330 (57.3)	226 (46.6)
	No	499 (47.0)	244 (42.4)	255 (52.6)
HIV	Yes	323 (30.4)	182 (31.6)	141 (29.1)
	No	738 (69.6)	394 (68.4)	344 (70.9)

^a^Behaviour during the 6-month period prior to interviews

Of the 1061 participants, 576 (54.3%) indicated a willingness to wear a device. As shown in Table 2, socio-demographic, behavioural and drug characteristics associated with a willingness to wear a device in unadjusted analyses included: female gender, ever overdosed, homelessness, methadone treatment, and chronic pain (all *p* < 0.05).

**Table 2:** Multivariate analysis of factors associated with the willingness to wear an overdose detection device (n = 1061)

Variables	Odds ratio (95% CI)	*P*	Adjusted odds ratio (95% CI)	*P*
Ethnicity	0.93 (0.73–1.19)	0.565		
Female	1.50 (1.17–1.94)	0.002	1.41 (1.09–1.84)	0.010
Age (years)	1.01 (1.00–1.02)	0.061		
Daily heroin use^a^	1.27 (0.98–1.65)	0.072		
Daily cocaine use^a^	0.95 (0.48–1.87)	0.872		
Daily crack use^a^	1.06 (0.70–1.60)	0.791		
Ever overdosed	1.41 (1.08–1.84)	0.011	1.39 (1.06–1.83)	0.017
Homelessness^a^	0.64 (0.48–0.85)	0.002	0.67 (0.50–0.91)	0.009
Methadone treatment^a^	1.94 (1.51–2.49)	< 0.001	1.86 (1.45–2.40)	< 0.001
Chronic pain	1.53 (1.20–1.95)	< 0.001	1.53 (1.19–1.96)	< 0.001
HIV	1.13 (0.87–1.47)	0.373		

*CI* confidence interval

^a^Behaviour during the 6-month period prior to interviews

The results of the multivariable analysis are presented in Table 2. As shown here, factors independently positively associated with willingness included ever having overdosed (adjusted odds ratio [AOR] = 1.39, 95% Confidence Interval [CI]: 1.06–1.83), current methadone treatment (AOR = 1.86, 95% CI 1.45–2.40), female gender AOR = 1.41, 95% CI 1.09–1.84) and a history of chronic pain (AOR = 1.53, 95% CI 1.19–1.96). Homelessness (AOR = 0.67, 95% CI 0.50–0.91) was negatively associated with willingness.

## Discussion

In the present study, just over half the participants interviewed were willing to wear an overdose detection device. In multivariable analyses, ever having overdosed, current methadone treatment and a history of chronic pain were positively associated with willingness, whereas homelessness was negatively associated with willingness.

We found that certain risk factors for overdose were associated with a willingness to wear a device. For instance, we have previously shown that non-fatal overdose is a risk factor for subsequent fatal overdose and it is interesting that a history of overdose was associated with willingness to wear a device [14]. Other well described risk factors for overdose, and a potential future areas to explore wearable technology, include recent release from prison, relapse after residential treatment, and not being on opioid agonist medication, like methadone [15, 16]. Supervised consumption sites and take home naloxone are well described interventions shown to prevent overdose deaths [17]; however, in recent years, in British Columbia, Canada unintentional overdose deaths have skyrocketed despite greater availability of these harm reductions interventions [18]. There is little debate they have prevented many overdose deaths, however, deaths in BC occur mostly in those using drugs alone who are not accessing these services [2, 3] and innovation is needed to prevent deaths in this population. Future research should seek to examine the population of individuals who indicated they were unwilling to wear a device. In some cases, this may be due to real or perceived low risk of overdose (e.g. currently abstinent). In other cases, it may be due to stigma and distrust reflecting the unwillingness of persons who use drugs to have information broadcast to first responders or others and when drug use remains criminalized.

Wearable technology has the potential to engage with people using drugs alone and potentially automatically call for help via cell phone technology or even automatically-administer naloxone to reverse overdoses. For example, a device being developed by Purdue University deploys a wristwatch-like device to measure respiratory rate and heart rate, surrogates for impending overdose [4]. It is logical that a device like this could communicate with other technology such as a cell phone to notify emergency services of an overdose or a even a “naloxone pump” [4].

Several next steps in this area of public health are needed. First, effective devices must be identified and validated that can reliably detect signs of opioid toxicity. As noted above, a recent study using basic cell phone technology was able to identify respiratory depression, apnea, and gross motor movements associated with acute opioid toxicity [9]. Similarly, devices are being developed that may be able to not only detect overdoses, but also administer naloxone [19]. However, it will be important to have technologies validated in safe laboratory settings and then well conducted real world research to identify potential benefits but also rigorously assess for potential harms in terms of potential unintended consequences such as a false sense of security when any future device will likely have imperfect ability to detect overdose.

This study has limitations. As cited previously, our study sample was generated through street-based recruitment methods, generalizing our findings to other populations of injection drug users requires caution. However, it is noteworthy that the cohort demographics are similar to other local and international studies of injection drug users [20–23]. Secondly, as our outcome of interest was willingness to wear an overdose detection device, actual rates of willingness and successful integration of such devices will need to be trialed in real world settings. Further, our study may be subject to socially describe responding whereby participants said they would wear a device when in reality they would not. However, when effective devices become available, potentially using existing items (e.g. cell phones), rates of willingness may change. Finally, socially desirable responding can be concern in studies of marginalized populations [24]. Nevertheless, we have previously shown how feasibility questions, such as those used in the present study, can be highly valid and accurately predict subsequent health service utilization [25, 26].

## Conclusion

In conclusion, in the present study we found that over 50% of those surveyed would wear an overdose detection mobile device and that a range of factors associated with overdose in this setting, including past overdose, were positively associated with willingness. Since a substantial number of persons said they would not wear a device and some factors, such as homelessness may be a barrier, further research is needed to investigate explanations for unwillingness and to evaluate real world acceptability of a wearable overdose detection devices as this technology becomes available.

## Data Availability

The datasets generated during and/or analysed during the current study are available from the corresponding author on reasonable request.

## Abbreviations

ACCESS: AIDS Care Cohort to Evaluate Access to Survival Services
AIC: Akaike information criterion
AOR: adjusted odds ratio
ARYS: At-Risk Youth Study
CI: confidence interval
IQR: inter-quartile range
NIH: U.S. National Institutes of Health
THN: take home naloxone
VIDUS: Vancouver Injection Drug Users Study
